# Variations in Nuclear Number and Size in Vegetative Hyphae of the Edible Mushroom *Lentinula edodes*

**DOI:** 10.3389/fmicb.2019.01987

**Published:** 2019-09-04

**Authors:** Qi Gao, Dong Yan, Dan Wang, Shanshan Gao, Shuang Zhao, Shouxian Wang, Yu Liu

**Affiliations:** ^1^Institute of Plant and Environment Protection, Beijing Academy of Agriculture and Forestry Sciences, Beijing, China; ^2^School of Agriculture, Ludong University, Yantai, China

**Keywords:** *Lentinula edodes*, multinucleus, non-nucleated, homokaryons, heterokaryons, apoptosis

## Abstract

In basidiomycete fungi, the number of nuclei and their ploidy level per nucleus can vary tremendously among species; however, within species, nuclear number and ploidy levels are traditionally considered fixed in their vegetative hyphae. In the edible mushroom *Lentinula edodes*, the hyphae are classified as either monokaryotic or dikaryotic, with each monokaryotic hyphal cell containing one haploid nucleus, and each dikaryotic hyphal cell containing two haploid nuclei. The dikaryotic hyphae are the results of mating between two genetically distinct monokaryons with different mating types. In this study, we examined the nuclear number and size (a potential correlate to ploidy) of *L. edodes* mycelia throughout its vegetative growth. We found that the number of nuclei within individual hyphal cells varied widely from non-nucleated to uninucleated, dinucleated, and multinucleated. Additionally, different nuclei within the same cell appeared very different in size, with a maximum nucleus cross-sectional area of 4.94 μm^2^ and the minimum nucleus cross-sectional area at only 0.37 μm^2^. Moreover, as culture time increased, more cells appeared to be devoid of any nuclei, with transmission electron microscopy and terminal deoxynucleotidyl transferase dUTP nick-end labeling (TUNEL) assays of late-stage cultures showing autophagosomes fusing and dissolving the nuclei and resulting in a large number of TUNEL-positive DNA fragments in non-nucleated cells. These results indicated that non-nucleated cells were likely caused by autophagy and apoptosis-like activities within aging *L. edodes* hyphae.

## Introduction

The research model of the life history of most mushrooms is based on the research on *Coprinus cinereus*, which was introduced early as an object for studies on edible basidiomycete development mainly because of its relatively short life cycle (Kües, 2000). In most basidiomycete species, homokaryons are referred to as monokaryons, which form from a germinated basidiospore and contain a single haploid nucleus in each cell compartment (Nieuwenhuis et al., 2013). At fertilization, nuclei from a compatible mycelium migrate into the other mycelium, at which point they are incorporated to form a heterokaryon (referred to as a dikaryon) (James et al., 2008). Dikaryons contain two haploid nuclei and share a single cytoplasm for a period of time without undergoing nuclear fusion or meiosis (Gladfelter and Berman, 2009). To maintain the dikaryotic stage during cytokinesis, some fungal species form a clamp that connects two adjacent hyphal cells and facilitates the segregation of the two daughter nuclei (Fischer, 1999; Gladfelter and Berman, 2009); however, this model is not always accurate, as the primary mycelium of *C. cinereus* does not fulfill the strictest definition of a monokaryon (Kües, 2000). Nuclear staining of aerial mycelium from various strains revealed that in most cases, up to half of all hyphal cell segments contain not just one but two nuclei (Polak et al., 1997). However, throughout the period of hyphal vegetative growth, it remains unclear whether this form of the nucleus is stable in *Lentinula edodes*. In previous studies, observations of nuclear behavior in edible fungi hyphae are mostly based on static observations at a certain time point. At different culture periods, nuclear behavior according to the number of nuclei in homokaryotic and heterokaryotic mycelia cells, as well as their variety in size, likely vary; however, there are few reports describing this.

*Lentinula edodes* (Berk.) Pegler is a tetrapolar basidiomycete and a major edible mushroom in Asia. Traditionally, the lifecycle of *L. edodes* is similar to that of most basidiomycete mushrooms. Each basidium produces four basidiospores that uninucleate through meiosis (Hasebe et al., 1991; Shimomura et al., 2011), after which the basidiospores germinate to grow into monokaryotic mycelium containing a single haploid nucleus in each cell compartment, with the cell separated by a septum. The dikaryon of *L. edodes* is typically distinguished by the following morphological characteristics: regular distribution of two haploid nuclei and formation of a clamp connection in each hyphal cell (Byeongsuk et al., 2017). The dikaryotic condition usually remains extremely stable during vegetative growth (Fukumasa-Nakai et al., 1994).

Here, we describe the nuclear number, size, and shape variations in *L. edodes* homokaryotic and heterokaryotic mycelia during different culture periods. In this study, we analyzed the processes involved in various nuclear behaviors and their respective influencing factors using electron microscopy. Our findings allow a deeper understanding of the lifecycle of higher basidiomycetes and clarify the variety in nuclear behavior during the vegetative-growth stage of *L. edodes* mycelium.

## Materials and Methods

### Fungal Strains

*Lentinula edodes* strains used in this study included 0912 and L808, which are widely cultivated in northern China. Fruiting bodies were collected from an *L. edodes* company in Beijing in June 2017, and tissues were isolated to obtain heterokaryotic strains. Strains were identified from the tissue isolates by antagonistic experiments, esterase isoenzyme experiments and simple sequence-repeat markers to confirm their species. The confirmed 0912 and L808 heterokaryotic strains were cultured on PDA medium at 25°C. Homokaryotic strains of 0912 and L808 were obtained by protoplast mononuclearization technology according to the procedure described by Fukumasa-Nakai et al. (1994). Heterokaryotic strains of 0912 and L808 were cultured potato dextrose broth in a shaking incubator at 25°C for 5 days. Cultured mycelia were treated with lywallzyme (Guangdong Microbial Culture Collection Center, Guangzhou, China) to obtain the protoplast, which were recovered on RM medium (1 M sorbitol in 4% PDA). Clamp connections in the recovered strains were observed with a light microscope (Olympus, BX51, Tokyo, Japan) to distinguish those without a clamp connection in order to identify homokaryotic strains. The confirmed 0912 and L808 homokaryotic strains were subcultured on PDA medium at 25°C for later use.

### Detection of Nuclei Number

Modification in MMP was used to determine time node by dyeing with an MMP assay kit (JC-1, Beyotime Biotechnology, Beijing, China). According to the results, the homokaryotic and heterokaryotic strains of 0912 and L808 were inoculated on slides with 0.2% glucose and 1% agar to inhibit aerial hyphae growth, and cultured for 11, 22, and 33 days to observe variations in nuclei number. Nuclear staining of mycelia was performed as previously described by Sawada et al. (2014). Briefly, mycelia were fixed in anethanol: acetic acid (3:1; v/v) solution for 20 min at room temperature and stained with (DAPI; Sigma-Aldrich, St. Louis, MO, United States) and Calcofluor White stain (Sigma-Aldrich) and observed under a fluorescence microscope (Olympus, IX71, Tokyo, Japan). In addition to fluorescent staining, the heterokaryotic strains were stained with Giemsa dye (Solarbio, Beijing, China) according to the method described by Dias et al. (2008). The heterokaryotic strains were fixed in 1 M HCl at 60°C for 10 min and washed in phosphate buffer before staining with 3% (w/v) Giemsa dye for 30 min.

Hundred apical cell compartments were selected from different hyphae on a slide. Subsequently, posterior cells were selected from different hyphae; these were all separated from the apical cell by 4–5 cells. In addition, three slides were selected as repetitions for each culture period. The overall experiment was repeated three times to ensure parallel accuracy of the experiment. Nuclei-number data were analyzed using Excel 2016 (Microsoft, United States), and each experiment was performed in triplicate.

### Transmission Electron Microscopy (TEM)

The morphologies associated with various nuclear phenotypes in different culture periods were observed by TEM. Heterokaryotic strains were cultured on PDA medium for 25, 50, and 75 days, respectively, and mycelium blocks were cut into 1 × 1 mm pieces, fixed with 2.5% glutaraldehyde in phosphate buffer (pH 7.2), dehydrated with an ethanol gradient, and embedded in Epon 812 resin (Electron Microscopy Sciences, Hatfield, PA, United States). Thin sections (70 nm) were cut with a diamond knife (Leica, EM UC7, Germany) and stained with 2% uranyl acetate for 10 min, followed by 3% lead citrate for 3 min. The samples were observed using a transmission electron microscope (JEOL JEM-1OOCX IL; JEOL, Tokyo, Japan) at 80 kV.

### Terminal Deoxynucleotidyl Transferase dUTP Nick-End Labeling (TUNEL) Assays

Terminal deoxynucleotidyl transferase dUTP nick-end labeling assays were performed using similar methods to those used for detecting the number of nuclei. Heterokaryotic strains were cultured on slides for 11, 22, and 33 days, fixed with 2.5% glutaraldehyde for 60 min, and stained using a TUNEL assay kit (Beyotime Biotechnology, Beijing, China) for 1 h. Stained mycelia were then washed with phosphate buffer and stained with DAPI for 60 min. Total nuclei and DNA fragments were observed in ultraviolet and green-fluorescence channels using a fluorescence microscope (Olympus). In quantitative analysis, to compare the TUNEL-positive with DAPI-positive nuclei numbers, a total of 300 hyphal cells were examined during each culture period, and each experiment was performed in triplicate.

### Image Data Analysis

Images analyses and measurements were performed using ImageJ software (National Institutes of Health, Bethesda, MD, United States). The calculation of nuclear volume and cytoplasm volume was performed according to the method of Neumann and Nurse (2007). Cells were measured by hand assuming the geometries with ImageJ, following Schneider et al. (2012), and volumes were calculated based on axial symmetries. The value of nuclear volume with cytoplasm volume (N/C) was calculated by the following formula:

N/C=N⁢u⁢c⁢l⁢e⁢a⁢r⁢v⁢o⁢l⁢u⁢m⁢e×N⁢u⁢c⁢l⁢e⁢a⁢r⁢n⁢u⁢m⁢b⁢e⁢r÷C⁢e⁢l⁢l⁢v⁢o⁢l⁢u⁢m⁢e

In each cell compartment, every nuclear N/C value was calculated and compared with its cross-sectional area to analyze the correlation between them. Data were analyzed by ANOVA. The significant differences between means were determined by Duncan’s multiple range test. Unless otherwise stated, the differences were considered statistically significant when *p* < 0.05, or extremely significant when *p* < 0.01.

## Results

### Nucleus Phenotype in Homokaryotic and Heterokaryotic Mycelia of *L. edodes*

**FIGURE 1:**
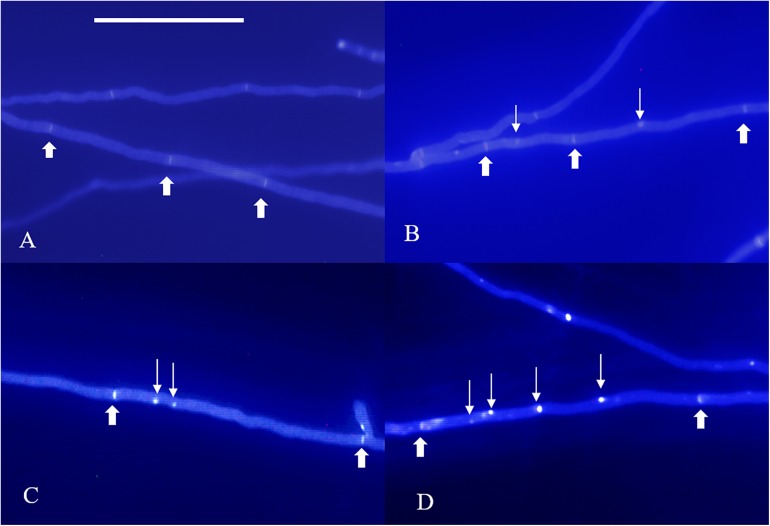
Nuclear phenotype of DAPI-stained *L. edodes* homokaryotic hyphae on the 11th day. **(A)** The non-nucleated, **(B)** uninucleated, **(C)** dinucleated, and **(D)** mutlinucleated phenotypes. Thick arrowheads indicate simple septa, and thin arrowheads indicate nuclei. Bar = 50 μm.

**FIGURE 2:**
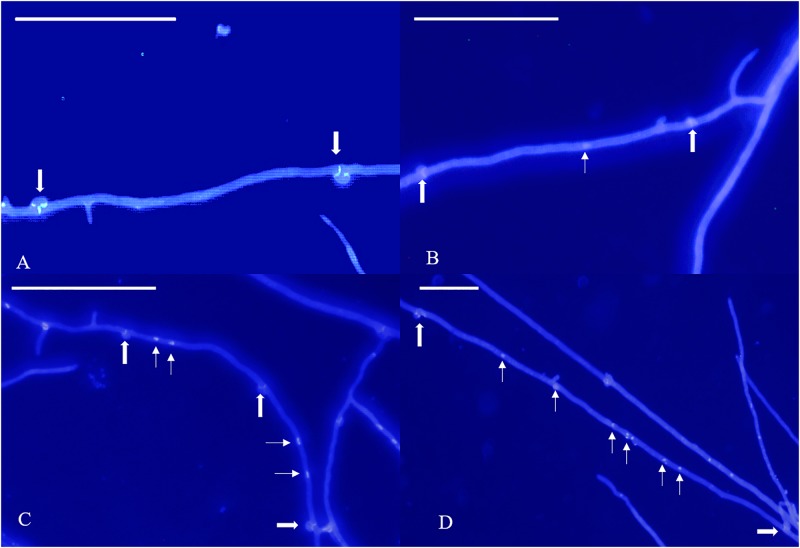
Nuclear phenotype of DAPI-stained *L. edodes* heterokaryotic hyphae on the 11th day. **(A)** The non-nucleated, **(B)** uninucleated, **(C)** dinucleated, and **(D)** mutlinucleated phenotypes. Thick arrowheads indicate clamp connections, and thin arrowheads indicate nuclei. Bar = 50 μm.

**FIGURE 3:**
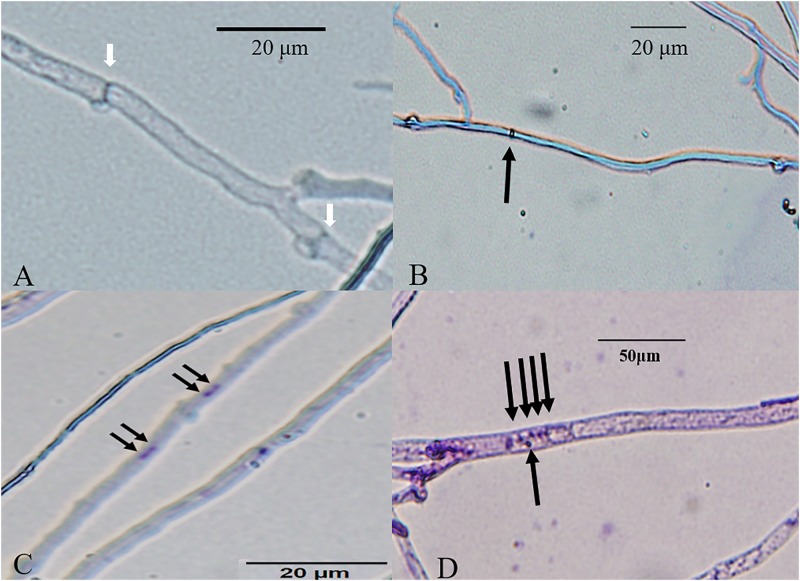
Bright-field micrographs of Giemsa-stained *L. edodes* heterokaryotic hyphae on 11th day. **(A)** The non-nucleated, **(B)** uninucleated, **(C)** dinucleated, and **(D)** mutlinucleated phenotypes. White arrowheads indicate clamp connections, and black arrowheads indicate nuclei.

According to our observations, homokaryotic hyphae were separated by a septum, and heterokaryotic hyphae were separated by clamp connections. However, different from traditional observations, both homokaryotic and heterokaryotic 0912 strains presented four nucleus-number phenotypes, including non-nucleated, uninucleated, dinucleated, and multinucleated phenotypes (Figures 1, 2). To eliminate the influence of DAPI dye, heterokaryotic 0912 strains were stained with Giemsa dye to observe nucleus numbers by light microscopy, the results of which confirmed the presence of the four different phenotypes (Figure 3). Additionally, homokaryons and heterokaryons of strain L808 exhibited four nuclear phenotypes (Supplementary Figure S1). Moreover, we collected a total of 10 strains of 0912 and L808 from different producing areas to repeat experiments, and observed a similar phenomenon. These results indicated the presence of non-nucleated and multinucleated phenotypes along with uninucleated and dinucleated cells in the homokaryotic and heterokaryotic hyphae of *L. edodes*, with this phenomenon observed in multiple *L. edodes* strains.

### Imbalances in Nuclear Sizes and Shapes

**FIGURE 4:**
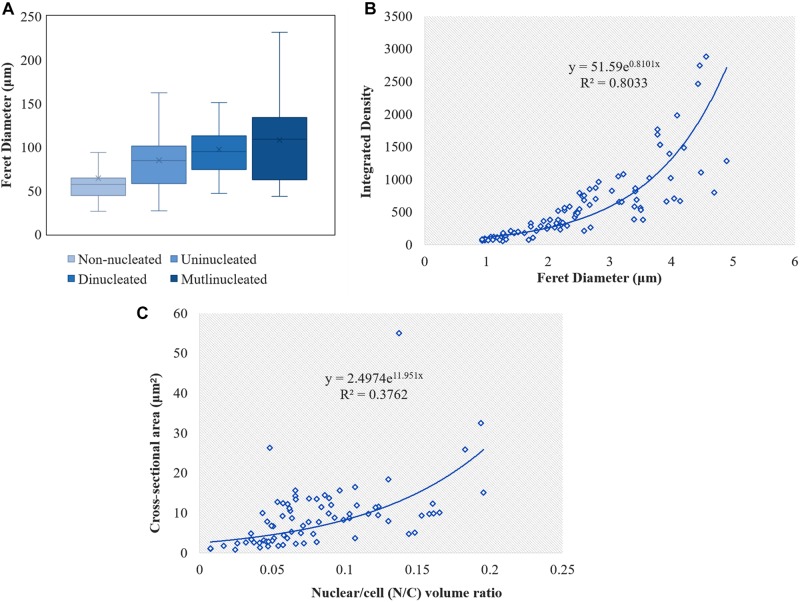
Calculation of the cell and nuclei size in *L. edodes* heterokaryotic hyphae. **(A)** Feret diameters of cells with different nuclei number. **(B)** Correlation between nucleic acid content and nucleus Feret diameter. **(C)** Correlation between N/C volume ratio and nuclear cross-sectional area.

**FIGURE 5:**
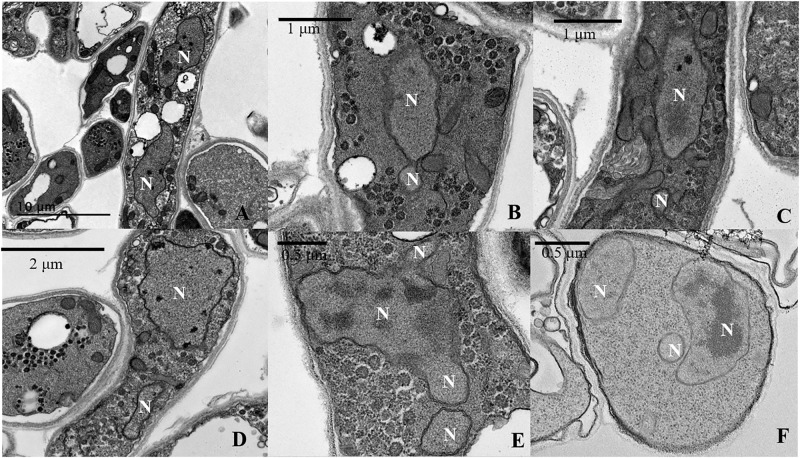
Transmission electron microscopy (TEM) analysis of imbalanced nuclear size and shapes in *L. edodes* heterokaryotic hyphae. **(A)** Balanced size of dinucleated cells. **(B)** Separation of a small nucleus from a large nucleus. **(C,D)** Imbalanced nuclear size. **(E)** Continuous separation of small nuclei from a large nucleus. **(F)** Imbalanced nuclear size in multinucleated heterokaryotic hyphae cells. N, nucleus.

We observed that the Feret diameter of cell varied significantly according to nucleui number (Figure 4A). The average cell Feret diameter for the multi-nucleated phenotype was the highest (107.9 μm) and nearly 2-fold highest than that of cells exhibiting a non-nucleated phenotype (64.1 μm). Additionally, nucleui number per cell differed significantly in cells with a multi-nucleated phenotype, with the largest heterokaryotic hyphae cell containing 47 nuclei. Moreover, the size of nuclei in the same cell was also significantly different (Figures 2D, 5). The maximum nucleus cross-sectional area was 4.94 μm^2^, and the minimum nucleus cross-sectional area was only 0.37 μm^2^. Even in cells with a dinucleated phenotype, the sizes of the two nuclei were not uniform and differed by a factor of up to 2.5.

We used the amount of fluorescence scattering from the nucleus following DAPI staining to reflect the nucleic acid content in different cells and analyzed this using ImageJ software. The results indicated a significant difference in nucleic acid content according to nucleus size, with correlations between nucleic acid content and nucleus size in heterokaryotic hyphae showing an exponentially positive relationship (Figure 4B). Additionally, we found differences in the N/C, with no correlations found between the N/C ratio and nucleus size in *L. edodes* (Figure 4C).

As per the TEM results, out of the observed 458 cells with nuclei in heterokaryotic hyphae, 120 cells belonged to dinucleated and multinucleated types. Nearly 70% of these cells exhibited an imbalanced size and shape of the nucleus in the same compartment (Figures 5B–F). Moreover, in situations where two nuclei in the same cell were similarly sized, the composition of the substances in the nuclei differed. Figure 5A shows that in the same cell, one of the of the nuclei contained a nucleolus and the other contained only heterochromatin, with neither observed in small nuclei (Figures 5B–F).

### Different Nucleus Phenotypes in Different Culture Periods

For the selection of the starting time, we observed the hyphae of *L. edodes* for 7, 9, and 11 days on the slide culture. The hyphae of *L. edodes* grew slowly on the slide culture. On the 11th day, the hyphae reached the apex of the slide. No significant differences was been observed in nuclear numbers on days 7, 9, and 11. Moreover, on the 11th day, the MMP exhibited red fluorescence by JC-1 dying without any depolarization. The determination of the time node thereafter was selected according to the change in MMP. On the 22nd day, the MMP began to decrease, and the staining result of JC-1 showed equivalent red and green fluorescence. On day 33, the result showed no JC-1 polymer aggregation in the mitochondrial matrix, and all of them showed a single-color morphology and showed only green fluorescence (Supplementary Figure S3). According to these results, we selected days 11, 22, and 33 as early, middle, and last stage of cultivation time node.

**FIGURE 6:**
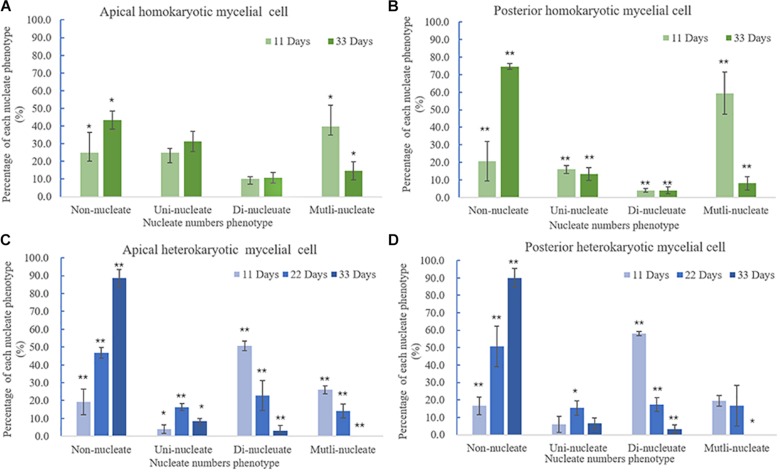
Variations in nuclei per cell according to culture time. **(A)** Apical and **(B)** posterior homokaryotic hyphae cells. **(C)** Apical and **(D)** posterior heterokaryotic hyphae cells. The error bars represent standard deviation. ^∗^*p* < 0.5 vs. culture time; ^∗∗^*p* < 0.01 vs. culture time.

The morphology of DAPI-stained cells on days 22 and 33 was similar to that of the cells on the 11th day (Figures 1, 2). The homokaryotic and heterokaryotic hyphae of strain 0912 contained four nuclei for all the of culture time periods. However, we found significant variations in nucleus-number phenotypes between groups according to culture time. We divided homokaryotic and heterokaryotic hyphae into apical-cell and posterior-cell groups and counted their nuclei numbers separately. During the early stage of cultivation (11 days), the proportion of multinucleated cells in the apical- and the posterior-cell groups of homokaryotic hyphae was the highest (Figures 6A,B). As the culture period increased and on the 33rd day of cultivation, the proportion of non-nucleated cells in homokaryotic hyphae was the highest. Moreover, we found a significant difference in the non-nucleated to multinucleated ratios in homokaryons between 11 and 33 days, with this difference more pronounced in the posterior-cell group. Furthermore, compared with the apical-cell group, the posterior-cell group of homokaryotic hyphae showed a higher proportion of non-nucleated cells during the early stage and a higher proportion of multinucleated cells during the late stage of cultivation. During all culture periods, the percentage of uninucleated cells of homokaryotic hyphae remained at <35%.

**FIGURE 7:**
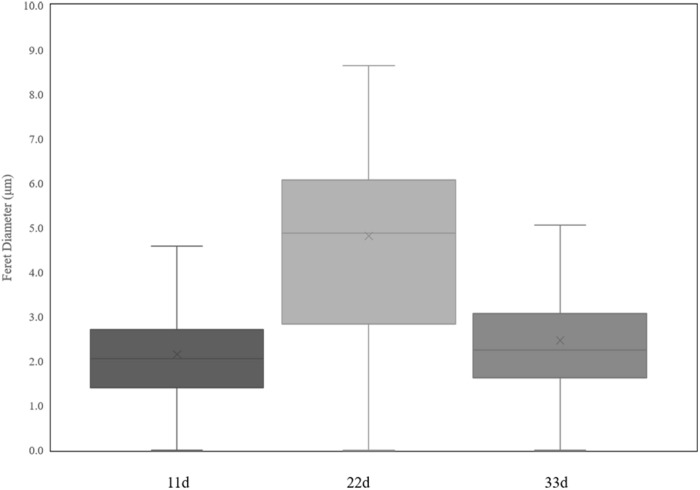
Variations in nuclei Feret diameter according to cultural time.

Heterokaryotic hyphae exhibited similar variations in nucleus number as homokaryotic hyphae, with dinucleated and multinucleated phenotypes more pronounced during the early stage of cultivation (Figures 6C,D). On the 22nd and 33rd days of cultivation, the number of non-nucleated cells in heterokaryotic hyphae increased significantly, reaching 50% in the posterior-cell group and >85% in both the apical- and posterior-cell groups, respectively. At the end of the culture, no multi-nucleated phenotype was observed in groups of heterokaryotic hyphae. The number of dinucleated cells in heterokaryotic hyphae was relatively high (50%) during the early stage while dropping to <5% in both groups at the end of the culture period. Additionally, analysis of variations in nucleus size in heterokaryons at different culture periods revealed that increasing culture time was accompanied by initial increases in the average size of the nuclei, followed by decreases in size (Figure 7). After 11 days of culture, the average Feret diameter of nuclei was 2.2 μm, and several small nuclei with a Feret diameter <1 μm and cross-sectional area <0.5 μm^2^ were observed. At 22 days, the average Feret diameter of nuclei was subsequently increased to 4.9 μm; however, at 33 days, small nuclei were rarely observed, and the average Feret diameter of nuclei decreased to 2.7 μm. Similar results were observed in homokaryotic and heterokaryotic hyphae of strain L808 strain (Supplementary Figure S2). These results confirmed the presence of this phenomenon across species and suggesting a significant difference from traditional observations.

### Apoptosis-Like Processes in Nuclei

**FIGURE 8:**
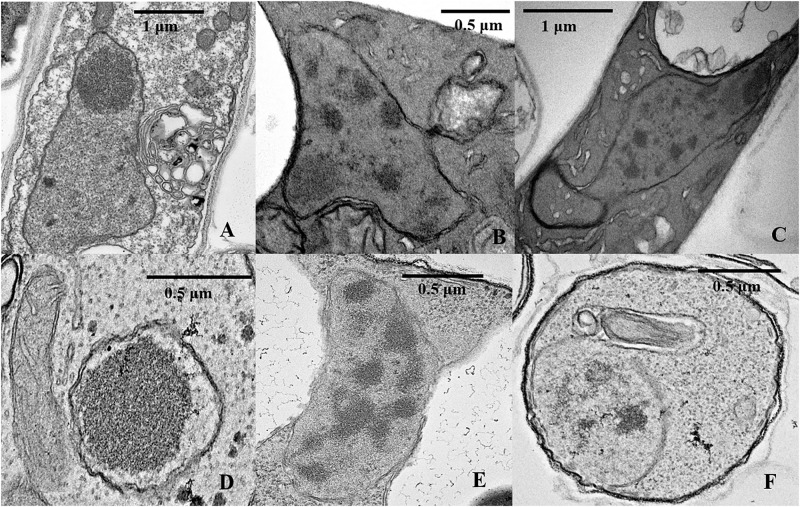
TEM analysis of nuclear characteristics according to culture time. **(A)** Normal intercellular-phase nucleus after a 25-day culture. **(B,C)** Nuclear characteristics after a 50-day culture. **(D–F)** Nuclear characteristics after a 75-day culture.

To further investigate the mechanisms associated with the significant increases in the number of non-nucleated cells according to increased culture time, TEM analysis and TUNEL staining of heterokaryotic hyphae cells were performed during different culture periods. Because PDA contains a higher degree of nutrients than slide culture, heterokaryotic hyphae cultured on PDA plates exhibited slower aging along with differences in other age-related phenotypes relative to those cultured on slides. After 25 days of cultivation, plates with heterokaryotic hyphae of *L. edodes* were completely covered, and aerial hyphae were enriched. After 50 and 75 days of cultivation, heterokaryotic hyphae exhibited phenotypes associated with progressive aging, and TEM analysis revealed that extended culture time resulted in increased indications of apoptosis-like activity in the nucleus. Additionally, on the 25th day of cultivation, interphase nuclei contained large, dense nucleoli (Figure 8A), with apoptosis-like features observed on the 50th day, including abnormalities in nucleus shape, increases in heterochromatin agglutination, disappearance of the nucleolus, and nuclear degeneration (Figures 8B,C). After the 75th day of cultivation, we observed further nuclear degeneration accompanied by nucleus solidification and shrinkage (Figure 8D), dissolving of the nuclear membrane by the vacuole (Figure 8E), and appearance of autophagosomes (Figure 8F), leading to autophagy of the nucleus.

**FIGURE 9:**
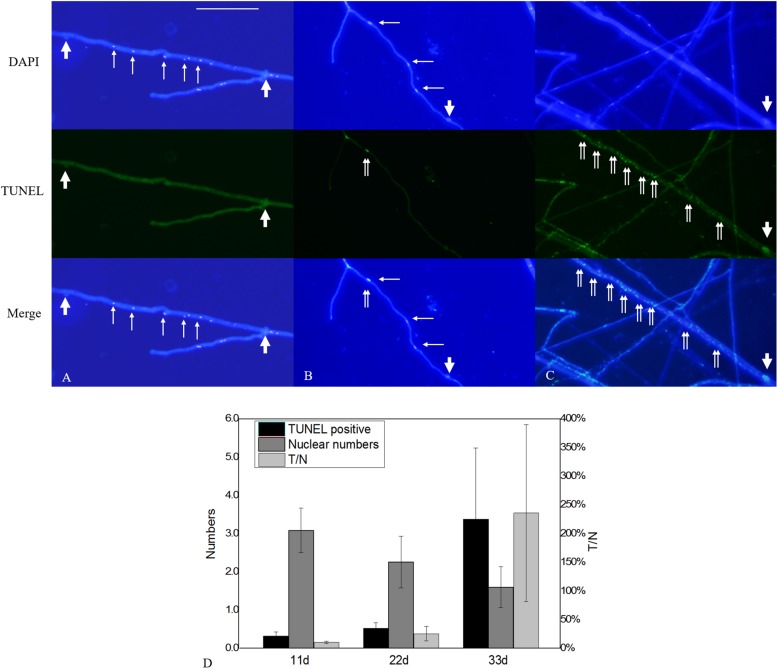
Nuclear and DNA-fragmentation phenotypes according to culture time. **(A)** Nuclear and DNA-fragmentation phenotypes on culture days 11, **(B)** 22, and **(C)** 33. **(D)** Quantitative analysis of TUNEL-positive and DAPI-positive nuclei numbers according to culture time. The error bars represent standard deviation. Thick arrowheads indicate clamp connections, and thin arrowheads indicate nuclei. Double arrowheads indicate DNA fragmentation. Bar = 50 μm.

**FIGURE 10:**
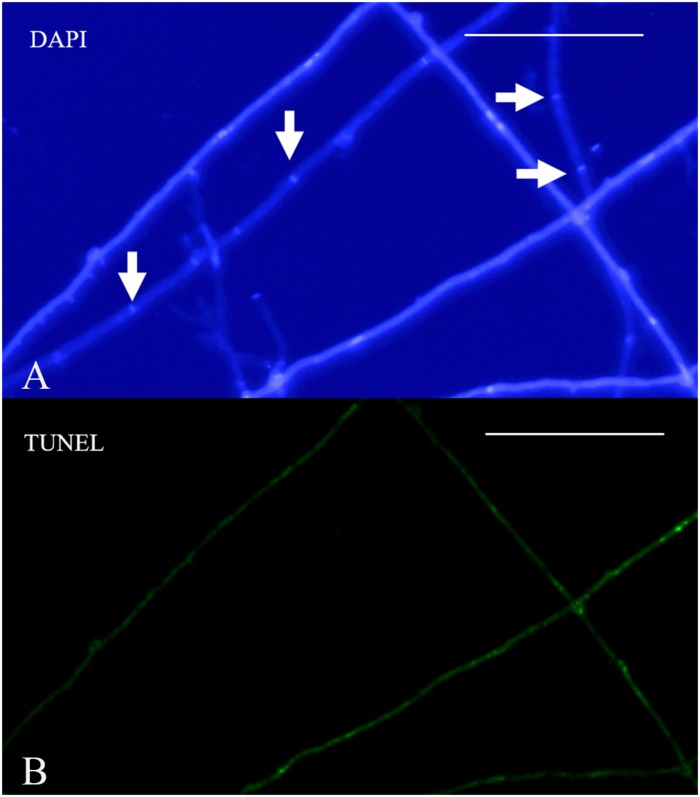
Nuclear and DNA-fragmentation phenotypes on culture day 33. **(A)** DAPI-stained heterokaryotic mycelia. **(B)** TUNEL staining showing DNA fragmentation. Arrowheads indicate septa. Bar = 50 μm.

**FIGURE 11:**
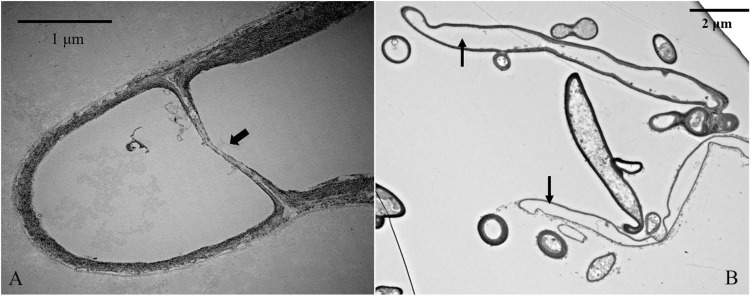
TEM analysis of organelle-free heterokaryotic hyphae cells. **(A)** Replacement of clamp connections with a septum. **(B)** No organelles were present in cells. Thick arrowhead indicates a septum. Thin arrowheads indicate empty hyphae cells.

Terminal deoxynucleotidyl transferase dUTP nick-end labeling assays showed that increases in culture time resulted in increases in TUNEL-stained DNA fragments. On the 11th day of slide culture, hyphae showed a multi-nucleated phenotype, with few TUNEL-stained DNA fragments (Figure 9A), whereas on the 22nd day, we observed reduced multi-nucleation and increased di-nucleation accompanied by increased TUNEL staining and weaker DAPI signals (Figure 9B). With respect to quantitative data, the TUNEL-positive nuclei number was 0.3 per cell, and the ratio of TUNEL to DAPI positive nuclei number was 10% (Figure 9D). On the 22nd day, the ratio rose to 30% (Figure 9D). These results indicated the increased presence of DNA fragmentation in the nucleus at this time in the presence of an intact nuclear structure. On the 33rd day, we observed a marked increase in non-nucleated cells accompanied by increased DNA fragmentation and TUNEL staining (Figure 9C), and the ratio of TUNEL to DAPI positive nuclei number increased to 236% (Figure 9D). Additionally, on culture day 33, we observed degradation of clamp connections, and septa were observed in heterokaryotic hyphae according to optical, fluorescence, and electron microscopy (Figures 10, 11A). This phenomenon occurred in both apical and posterior cells in the hyphae, with the cells becoming shorter than those with an intact clamp connection. Moreover, DAPI and TUNEL staining revealed the absence of nuclei and DNA fragments in the cells (Figure 10), with no organelles identified according to TEM analysis (Figures 11A,B).

## Discussion

In this study, we observed morphological changes in nuclei in homokaryotic and heterokaryotic hyphae of *L. edodes*, revealing simultaneous existence of four kinds of nucleated phenotypes (non-nucleated, uninucleated, dinucleated, and mutlinucleated) exhibiting significant variations in nucleus number according to culture time. During the initial stage of cultivation, the *L. edodes* mycelium grows rapidly, and both the homokaryotic and heterokaryotic hyphae showed a mutlinucleated or dinucleated phenotype. In *Fusarium oxysporum*, the number of mycelial cells is positively correlated with mycelial growth rate, with hyphae harboring a high number of nuclei exhibiting rapid growth (Shahi et al., 2015), which plays a role as a nutrient scout. A similar phenomenon was observed in *Neurospora crassa*, where fast-growing apical compartments allow maintenance of a specific nuclear density. This trait of homokaryotic hyphae is similar to subnormal behavior first reported by Boidin (1971); however, there have been no reports of changes in the number of heterokaryotic mycelial nuclei from a multinucleated phenotype to a dinucleated then non-nucleated phenotype over time.

With the exception of a small number of aging mycelial cells, clamp connections can be observed in non-nucleated, uninucleated, or multinucleated cell of heterokaryons. Additionally, multinucleated cells have been found in *C. cinereus* and *Agaricus brasiliensis* dikaryons (Kües, 2000; Dias et al., 2008). These findings suggest that nuclear behavior is more complicated than previously reported, and that clamp function is not essential for stable and accurate dikaryon formation (Gladfelter and Berman, 2009). Nuclear migration is mainly mediated by the actin cytoskeleton and microtubules, which through subsequent nuclear positioning (Nakai and Ushiyama, 1978; Tanabe and Kamada, 1994) and regulating spindle length and alignment might be the basis for alternating genotypes to establish dikaryons (Gladfelter and Berman, 2009).

During observation of mutlinucleated phenomena in heterokaryotic hyphal cells, we also observed that these cells were often accompanied by imbalanced nucleus size within the same cell. Moreover, dinucleated cells also showed a non-uniform size in the two nuclei, potentially differing by 2.5-fold. Previous studies proposed a nucleoskeletal theory suggesting the influence of DNA content on nuclear volume (Cavalier-Smith, 2005; Gregory, 2005). Subsequent experiments in mice supported this theory, revealing that tetraploid mouse embryos harbored nuclei twice as large as those in a diploid control (Henery et al., 1992; Henery and Kaufman, 1992). However, in a report studying fission yeast (*Schizosaccharomyces pombe*), nucleus size was independent of the amount of DNA, with a 16-fold change in nuclear DNA content having no influence on the relative size of the nucleus, which was related to the cytoplasm (i.e., a large cytoplasmic space allowed formation of a large nucleus) (Neumann and Nurse, 2007; Roberts and Gladfelter, 2016). In the present study, nucleus size in *L. edodes* was positively correlated with DNA content, and we observed no correlation between N/C volume ratio and nucleus size. Therefore, these results in *L. edodes* differed substantially from those in yeast in reference to the regulation of nucleus size.

Some nuclear behavior in *L. edodes* might mimic that in mammalian cells. TEM analysis revealed small nuclei splitting from the large cell nucleus, with this phenomenon similar to the micronucleus of cancer cells. Micronuclei result from either chromosome breakage or imperfect mitosis, when a chromosome fragment or an entire chromosome becomes separated from the bulk of the DNA (Webster et al., 2009). In *L. edodes* mycelial cells, small nuclei split from the large nucleus displayed weak fluorescence and almost no heterochromatin was observed by TEM. Similarly, the formation of small nuclei (termed “micronuclei”) was observed in mature heterokaryotic hyphae of *F. oxysporum* (Shahi et al., 2016). Another study reporting micronuclei in *Trichoderma reesei* (Toyama and Toyama, 1995) observed their presence following treatment with high concentrations of colchicine, which produced a large number of small nuclei and indicated a significantly lower DNA content (∼35% lower) in the small cell nucleus relative to that in the normal cell nucleus. Additionally, micronuclei in plant and animal cells are often produced by various physical and chemical factors, such as radiation and chemical agents, acting on dividing cells (Webster et al., 2009). However, in the present study, we added no physical or chemical environment pressure, and the reason for the formation of the small nuclei remains unclear. Moreover, we found that the average nucleus size initially increased along with extended culture times of *L. edodes* heterokaryons, followed by a gradual decrease, and that the number of small nuclei decreased along with increased culture time. At the end of the culture, we observed very few small nuclei and a large number of non-nucleated cells and those lacking organelles, indicating that the mycelial cells regulated the number and size of nuclei over time.

As culture time increased, both apical- and posterior-cell groups of *L. edodes* homokaryotic and heterokaryotic hyphae gradually displayed a non-nucleated phenotype, with the rate of non-nucleation higher in posterior cells and no multinuclear phenotype observed in either group of heterokaryotic hyphae at the end of the culture. A single nuclear senescence gene mutant can occur in monokaryons, such as in *N. crassa* (Maheshwari, 2005). The senescence mutant nuclei can lead to the monokaryon dying in 2–4 subcultures; however, this phenomenon was masked in the heterokaryotic hyphae. Self-regulated autophagy or apoptosis-like activity might be an explanation for mycelium presentation of a non-nucleated phenotype. Under starvation conditions, the filamentous fungus *Aspergillus oryzae* is capable of degrading nuclei from compartments of older mycelium through macroautophagy and utilizing the released nutrients to support colony survival and growth (Shoji et al., 2010). Additionally, Fang et al. (2017) found that *Atg8* expression in *L. edodes* mycelium after brown-film formation was higher than that before this process, indicating that autophagy might participate in the color-transfer process. Brown-film formation has been described as a step in morphogenesis peculiar to *L. edodes* and usually found on the surface of the mature mycelium (Tang et al., 2013). In the present study, we observed formation of a large area of brown film after 75 days of culture. Moreover, during TEM analysis indicated autophagosome activity in close proximity to the nuclear envelope and involved in its dissolution in heterokaryotic hyphae of *L. edodes*. This suggested that the macroautophagy process in *L. edodes* had been induced, and that autophagic destruction of the nuclei was underway.

A programed nucleus-destruction process was proposed during the development the meiotic yeast spore (Eastwood et al., 2012). In this yeast gametogenesis, a conserved mega-autophagy collaborates with apoptosis-like activity to destroy uncellularized nuclei using vacuolar machinery. These results are more in line with our observations. In addition to observing the vacuolar erosion of the nuclear envelope, TUNEL staining of heterokaryotic hyphae cells of *L. edodes* revealed a large number of TUNEL-positive DNA fragments in the non-nucleated cytoplasm at the late stage of culture, indicating fragmentation of nuclear DNA by endonucleases and destruction of the intact nucleus. Subsequent to the apoptosis-like activity in *L. edodes*, large amounts of free fragmented DNA ultimately replaced the nuclei in a process involving vacuole machinery and similar to that observed in yeast programed nucleus destruction. Finally, the mycelial cells displayed only a cell wall in the absence of a cytoplasm or organelles, suggesting their transformation into structural mycelia and decomposition of internal substances for utilization by new hyphae.

In summary, our results clarified changes in the number of nuclei present in *L. edodes* homokaryotic and heterokaryotic hyphae from mutlinucleated to non-nucleated phenotypes according to increases in culture time. Additionally, TEM and TUNEL analyses revealed the presence of autophagy and apoptosis-like activities associated with formation of the non-nucleated phenotypes-like activities in *L. edodes* hyphae. Moreover, long-term culture of *L. edodes* hyphae suggested that they might mediate their rapid growth, reproduction, and/or nutrient redistribution by regulating the number of nuclei in order to better maintain the vitality and stability of the entire colony environment.

## Data Availability

The raw data supporting the conclusions of this manuscript will be made available by the authors, without undue reservation, to any qualified researcher.

## Author Contributions

SW, YL, and QG conceived and designed the study. QG, DY, DW, SG, and YL performed the experiments. QG, DY, and SW analyzed the data. YL, SW, QG, and SZ contributed reagents, materials, and analysis tools. QG, SW, and YL wrote the manuscript.

## Conflict of Interest Statement

The authors declare that the research was conducted in the absence of any commercial or financial relationships that could be construed as a potential conflict of interest.

## Supplementary Material

FIGURE S1 Nuclear phenotype in *L. edodes* homokaryotic and heterokaryotic hyphae of strain L808. Non-nucleated, uninucleated, dinucleated, and multinucleated phenotypes in (**A–D**, respectively) homokaryons and (**E–H**, respectively) heterokaryons. Thick arrowheads indicate simple septa or clamps and thin arrowheads indicate nuclei. Bar = 50 μm.

FIGURE S2 Variations in nuclei per cell according to culture time of *L. edodes* L808 heterokaryotic hyphae. **(A)** Apical hyphae cells. **(B)** Posterior heterokaryotic hyphae cells. ^∗^*p* < 0.5 vs. culture time; ^∗∗^*p* < 0.01 vs. culture time.

FIGURE S3 Modification in mitochondrial membrane potential according to culture time of *L. edodes* 0912 heterokaryotic hyphae. Mitochondrial membrane potential with JC-1 dyeing on culture days **(A)** 11, **(B)** 22, and **(C)** 33.

## Abbreviations

DAPI: 4′,6-diamidino-2-phenylindole dihydrochloride
JC-1: 5,5′,6,6′-tetrachloro-1,1′,3,3 ′-tetraethyl-imidacarbocyanine iodide
MMP: mitochondrial membrane potential
N/C: nucleus/cell volume ratio
PDA: potato dextrose agar
TEM: transmission electron microscopy
TUNEL: terminal deoxynucleotidyl transferase dUTP nick-end labeling.
