# Supplementary figures and images for: Variations in Nuclear Number and Size in Vegetative Hyphae of the Edible Mushroom Lentinula edodes
Source: Front Microbiol. 2019 Sep 4;10:1987. doi: 10.3389/fmicb.2019.01987 (PMC6737286; doi:10.3389/fmicb.2019.01987)

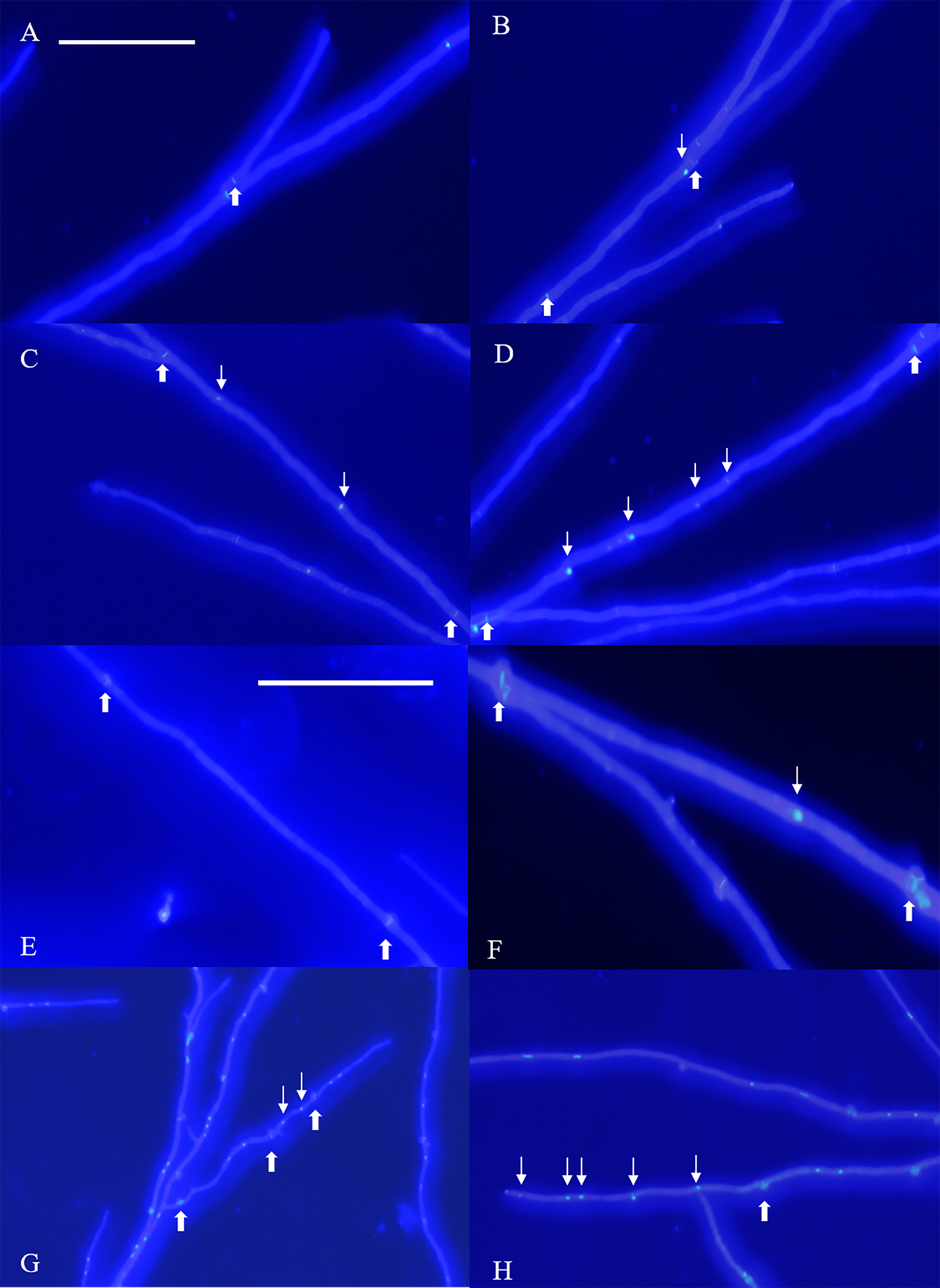

Supplement: FIGURE S1 — Nuclear phenotype in L. edodes homokaryotic and heterokaryotic hyphae of strain L808. Non-nucleated, uninucleated, dinucleated, and multinucleated phenotypes in (A–D, respectively) homokaryons and (E–H, respectively) heterokaryons. Thick arrowheads indicate simple septa or clamps and thin arrowheads indicate nuclei. Bar = 50 μm. [file Image_1.TIF]

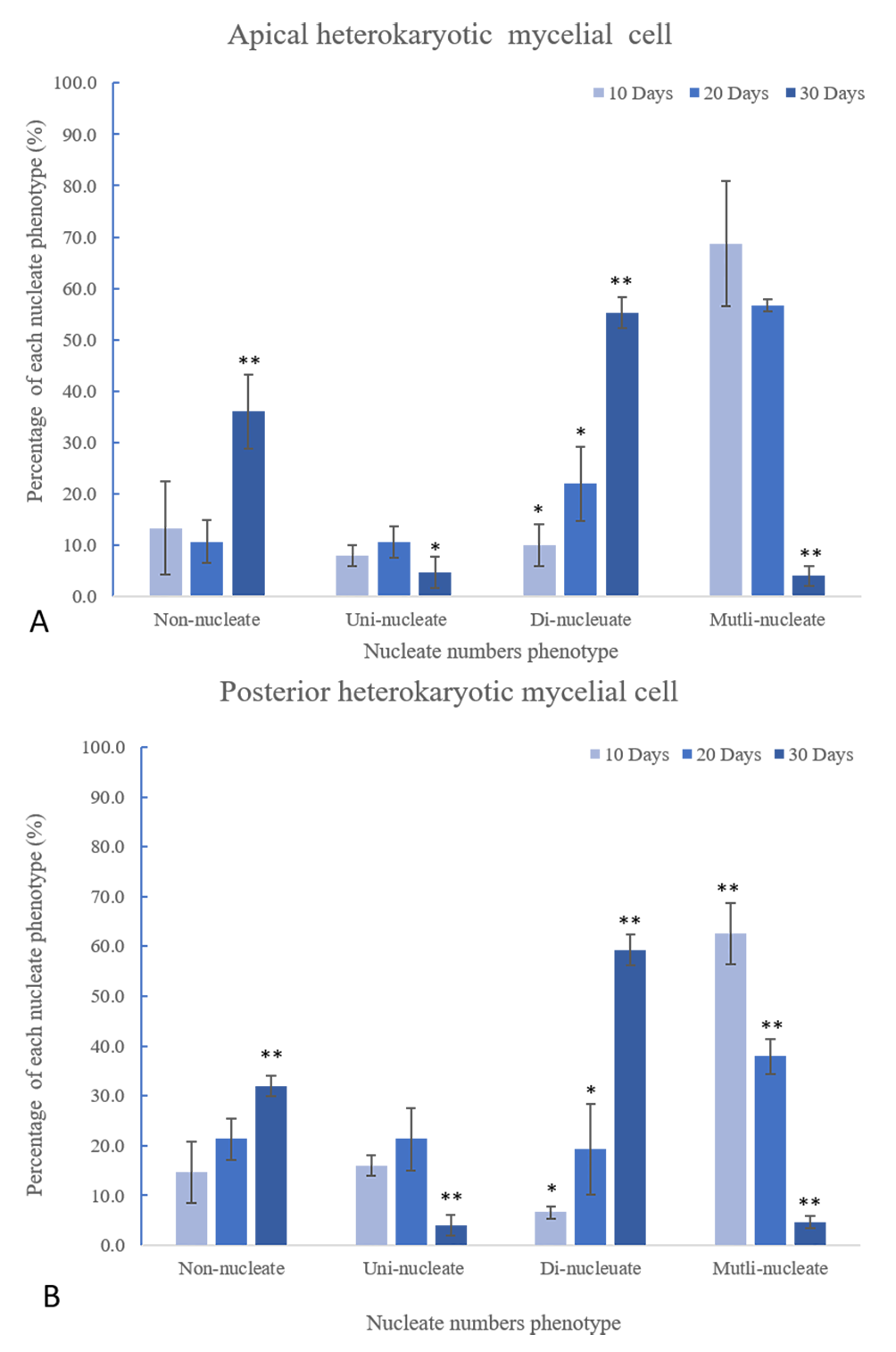

Supplement: FIGURE S2 — Variations in nuclei per cell according to culture time of L. edodes L808 heterokaryotic hyphae. (A) Apical hyphae cells. (B) Posterior heterokaryotic hyphae cells. ∗p < 0.5 vs. culture time; ∗∗p < 0.01 vs. culture time. [file Image_2.TIF]

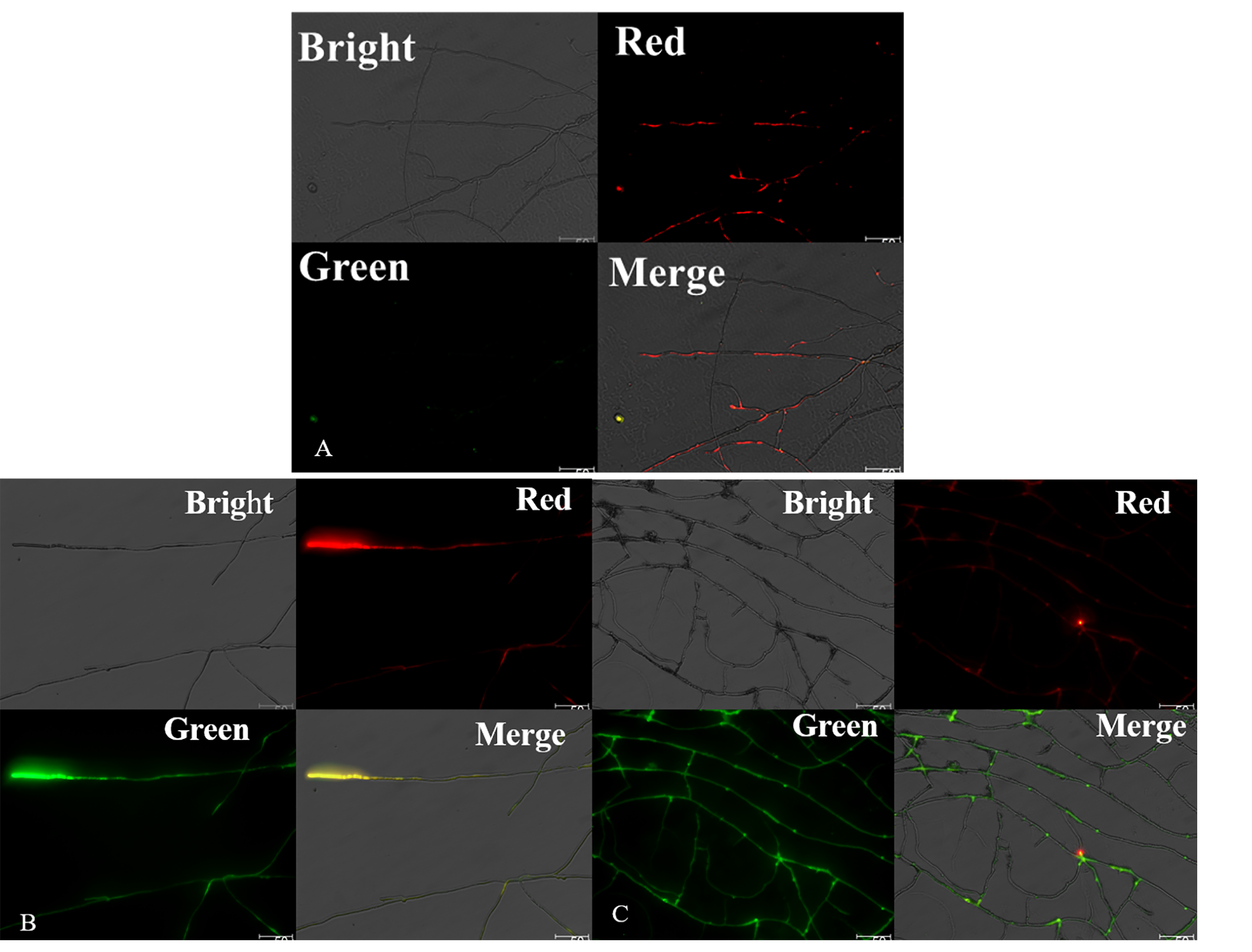

Supplement: FIGURE S3 — Modification in mitochondrial membrane potential according to culture time of L. edodes 0912 heterokaryotic hyphae. Mitochondrial membrane potential with JC-1 dyeing on culture days (A) 11, (B) 22, and (C) 33. [file Image_3.TIF]
